# Severe enduring anorexia nervosa (SE-AN) treatment options and their effectiveness: a review of literature

**DOI:** 10.1186/s40337-024-01006-y

**Published:** 2024-04-23

**Authors:** Federica Marcolini, Alessandro Ravaglia, Silvia Tempia Valenta, Giovanna Bosco, Giorgia Marconi, Diana De Ronchi, Anna Rita Atti

**Affiliations:** 1 Department of Biomedical and Neuromotor Sciences, University of Bologna, Viale Pepoli 5, 40123 Bologna, Italy; 2 Department of Clinical Nutrition, AUSL Bologna, Bologna, Italy; 3 U.O. Cure Primarie, AUSL Area Vasta Romagna, Ambito di Rimini, Rimini, Italy

**Keywords:** Anorexia nervosa, Eating disorders, Chronicity, Severe-enduring, Treatments

## Abstract

**Introduction:**

For nearly 20% of patients diagnosed with Anorexia Nervosa (AN), the eating disorder (ED) is prolonged and becomes long-lasting. It has been reported that patients diagnosed with Severe Enduring Anorexia Nervosa (SE-AN) have worse ED symptoms, higher rates of lifetime hospitalization, and lower psychosocial well-being compared to patients with shorter disease duration.

**Objectives:**

This review aims to describe the treatments proposed to date and their effectiveness on SE-AN-related outcomes.

**Methods:**

We conducted a PubMed search for studies addressing the issue of treatment approach to SE-AN adults, that were published between 2003 and 2023, peer-reviewed, written in the English language, and available in full-text. Next, we inductively created relevant macro-themes by synthesizing the data from the included articles.

**Results:**

Of 251 PubMed studies, 25 articles were considered for data extraction, all published between 2003 and 2022. We identified three macro-themes. The first macro-theme, “Psychotherapy”, mostly takes into consideration treatment effectiveness of cognitive behavioral therapy (CBT). Various reports determined its greater effectiveness compared to Specialist Supportive Clinical Management (SSCM), and one study proved that outpatient CBT is a valid alternative to hospitalization. The second one involves “Pharmacological Treatments”. Research on dronabinol, a synthetic orexigenic cannabinoid, antipsychotics (in particular, olanzapine and haloperidol), and ketamine showed some mixed results regarding the often-complementary areas of weight gain and improvement in ED-related symptoms. Regarding the third macro-theme, “Brain Stimulation Therapies,” such as Repetitive Transcranial Magnetic Stimulation (rTMS) and Deep Brain Stimulation (DBS), we found promising results in improving ED-related psychological traits (such as mood and anxiety), affective regulation, and quality of life. However, we have observed divergent results regarding outcome measures such as BMI and weight gain.

**Conclusions:**

SE-AN patients are predicted to encounter both medical complications and psychological distress of increasing severity that will inevitably affect their quality of life; to our knowledge, research evidence on treatment options for SE-AN remains limited, and the methodological quality of studies is generally low. These findings denote the need to focus future research efforts on effective treatment strategies specific to long-lasting EDs.

## Abstract (plain language summary)

For nearly 20% of patients diagnosed with Anorexia Nervosa, the eating disorder is prolonged and becomes long-lasting. Those patients have worse ED symptoms, higher rates of lifetime hospitalization, and lower psychosocial well-being compared to patients with shorter disease duration. This review aims to describe the treatments proposed to date and their effectiveness on severe enduring anorexia nervosa related outcomes. The data obtained show how the intervention techniques primarily used in these patients are psychotherapy (in particular, cognitive behavioral therapy and Specialist Supportive Clinical Management), pharmacological treatments, and Brain Stimulation Therapies (such as Repetitive Transcranial Magnetic Stimulation and Deep Brain Stimulation). To our knowledge, research evidence on treatment options for SE-AN remains limited and these findings denote the need to focus future research efforts on effective treatment strategies specific to long-lasting eating disorders.

## Introduction

About 50–80% of people diagnosed with Anorexia Nervosa (AN) have been estimated to achieve full or partial recovery [1, 2]*.* However, for nearly 20% of patients, the eating disorder (ED) is prolonged and becomes long-lasting [3, 4].

It is certainly true that AN patients who do not achieve a healthy weight and do not improve their ED psychopathology are predicted to encounter both medical complications and psychological distress of increasing severity, which will inevitably affect their quality of life [5–8]. Thus, according to Calugi et al., programs that simply target harm minimization and quality-of-life improvement, without emphasizing weight restoration, should be considered only for patients with AN who have been unsuccessful in recovery-based outpatient and inpatient treatments, and/or for those with persistent low motivation to change, in whom engagement procedures have repetitively failed, regardless of the duration of the illness [9].

Currently, there is no clear consensus on the definition of a severe and enduring eating disorder (SE-ED). The most common defining criteria for SE-ED involve disease duration and number of unsuccessful treatment attempts [10]*.* Compared with other EDs, Severe Enduring Anorexia Nervosa (SE-AN) has been better defined and more extensively studied [11–13].

In a recent study*,* it was reported that patients defined as SE-AN (characterized by high current distress and disease duration of at least seven years) had worse ED symptoms, higher rates of lifetime hospitalizations and lower psychosocial well-being compared to patients with shorter disease duration [14]. In addition, the SE-AN patient group was found to have poorer improvement in ED symptoms, BMI and work/social adjustment over time [14]. Furthermore, patients classified as SE-AN reported a higher rate of accessing intensive services, higher ED symptomatology, poorer work/social adjustment at baseline and lower rates of improvement in work/social adjustment at 12 months compared to "early stage" respondents [15–18].

Recently, treatment options for SE-EDs have been widely discussed, although research evidence on the topic remains limited and the methodological quality of the studies is generally low [19, 20]. According to the traditional approach, the first step in the treatment of EDs is to improve nutritional status, which is pursued before psychological treatment can be successfully implemented. In SE-ED, experts have suggested a different approach to treatment [19, 21, 22]. It has been proposed that the main treatment focus should be brought to quality of life and work/social adjustment rather than pursuing a significant reduction in ED symptoms [21, 23]*.* The rationale behind this proposal is to prevent challenging and demanding treatment targets from being overwhelming, promoting resistance to treatment and leading to counterproductive consequences among patients, including increased risk of suicide [11].

Severe food deprivation is an important maintenance factor in EDs. Long-term poor nutritional status and dysregulated eating behavior alter brain structure and function [24]*.* In view of this, patients with SE-ED could benefit from innovative treatment modalities, such as Deep Brain Stimulations (DBS) designed to rebalance the maintenance factors of the disorder [21].

Furthermore, although in EDs response to pharmacological treatments has been quite weak, especially in AN [25]*,* it has been suggested that patients with SE-ED could benefit from using some medications, especially those that induce weight gain, such as Olanzapine [25–27]*,* or those that stimulate appetite, such as Dronabinol, a cannabinoid drug [28–30]*.* However, even after months of treatment attempts, results on eating behaviors and weight gain have been poor.

In conclusion, current scientific evidence on SE-AN treatments is unfortunately still limited. The aim of this review is to describe the different treatments that have been proposed to date and their effectiveness on SE-AN-related outcomes.

## Materials and methods

This review was conducted in accordance with the Preferred Reporting Items for Systematic Reviews and Meta-Analyses Extension for Scoping Reviews (PRISMA-ScR) [31]. We conducted this literature review starting from the identification of keywords that allowed us to detect the various treatments proposed to date for SE-AN and their effectiveness, as described in the next section. We then applied a method of narrative synthesis of the data and divided the results into three macro-themes. The macro-themes were not defined a priori*,* but derived by induction from a process of analysis of the relevant literature. The included articles were summarized using text and tables. Ethical approval was not sought for the present study because it retrieved and synthesized data from previously published studies.

### Research strategy

Two authors (A.R. and F.M.) independently searched PubMed/MEDLINE databases from the 1st of March 2023 to the 31st of August 2023. Article research was conducted using the following search terms: eating disorder, chronic, refractory, resistant to medical treatment, failure of treatment, critical, severe, long-lasting, enduring AND Anorexia Nervosa. Further article searches were run by using the words: nutritional therapy, dietary treatment, pharmacological therapy, psychotherapy, cognitive therapy, behavioral therapy, and mindfulness. In light of the meager literature on the topic, keywords were searched in titles, abstracts and text, in order to include every possible therapeutic approach *cue*.

### Eligibility criteria

We included articles addressing the issue of treatment approach to SE-AN adults, published between 2003 and 2023, which were peer-reviewed, written in the English language, and available in full text. Articles were excluded if they involved non-target populations (non-adult, non-SE-AN populations), non-target topics (e.g., descriptive articles regarding EDs or SE-AN), and non-target designs (e.g., dissertations).

Preliminarily, the abstracts were screened for inclusion and exclusion criteria, and then selected articles were comprehensively examined in full text. In addition to keyword searching, we used citation chaining in full-text screening to intercept content that the original searches may have missed.

### Data extraction and management

**Fig. 1:**
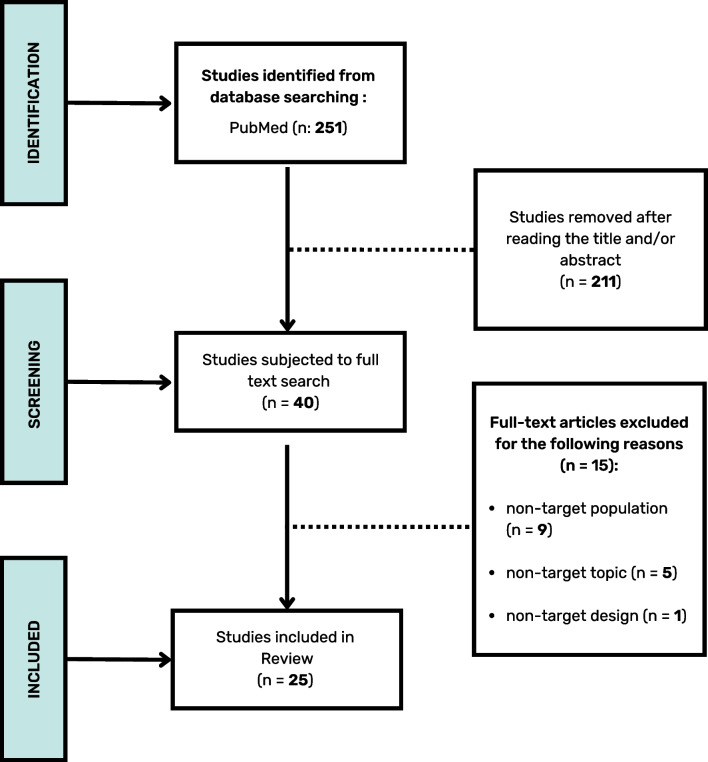
PRISMA flowchart

Two authors (A.R. and F.M.) independently extracted data from the eligible articles following this scheme: sample demographic information, treatment approach, and results. All relevant screened papers were collected using a dedicated Excel spreadsheet. A third reviewer (A.R.A.) resolved any discrepancies. Finally, a flowchart following the PRISMA guidelines was created to summarize the different phases of the selection process (Fig. 1)[31].

## Results

### Studies features

Twenty-five articles were considered for data extraction (Table 1**),** all published between 2003 and 2022. They were conducted in the UK, Norway, the United States, Australia, Italy, Israel, Denmark, Luxembourg, and Spain.

**Table 1:** Main features of the included articles

First author/ Year/ Country	Type of study	Treatment proposed	N. participants/ Diagnosis/ Mean BMI	Mean age/ Mean DD	Outcome measures	Main results
Dalle Grave 2013 Italy	RCT	**CBT**	N = 80 AN BMI:14.3	23,4 5	BMI	Most patients completed CBT, both groups showed improvements in weight and ED. No significant differences
Calugi 2017 Italy	Longitudinal trial	**CBT**	N = 66 SE-AN BMI: 14.7	26.1 7.7	BMI, EDE, GSI and BSI	Inpatient CBT-E achieved a significant increase in BMI, and substantial improvements in ED-specific and general psychopathology in both SE-AN and NSE-AN group of patients
Frostad 2021 Norway	Quality Assessment	**CBT**	N = 21 SE-AN < 16	25,5 7,3	BMI	At 1 year, 66.7% of patients had BMI > 18,5 kg/m2. Results» CBT-E as a valid alternative to hospitalization
Touyz 2013 Australia e UK	RCT	CBT SSCM	N = 63 SE-AN 16,2	33,4 16,6	Quality of life, Weight gain, ED symptoms	Both groups improved weight and EDE. CBT-AN had lower EDE scores at 12 months
Le Grange 2014 UK, Australia	RCT	CBT SSCM	N = 63 SE-AN BMI:16.2	3,0.4 16,6	EDQOL, MCS, BDI	CBT-AN was found to be more beneficial than SSCM for worse depression level, older age, more severe ED-related symptoms, AN-BP subtype
Elbaky 2014 Australia USA	RCT	CBT SSCM	N = 63 SE-AN BMI:16	NR 14	EDE, EDQOL, SF, ANSOQ	Those who did not complete treatment» had restrictive AN subtype and worse EDEQOL
Schwartz 2020 USA	Longitudinal trial	IM Ketamine	N = 4 AN or BN 17,5 < BMI < 37,8	36,7 > 7	BDI, EDE-Q, BMI	IM Ketamine effective for depressive aspects in TRD and AN patients. Minor positive results in reducing ED symptoms. Reduced risk of suicide
Andries 2014 Denmark	RCT	Dronabinol	N = 24 AN BMI NR Caucasian	> 18 > 5	Weight, EDI-2, Leptin levels	Dronabinol gave a small weight improvement. No significant differences between Dronabinol and Placebo in changing ED symptoms
Attia 2019 USA	RCT	Olanzapine	N = 152 SE-AN BMI: 16.8	29 11.55	BMI and weight, YBOCS, EDE, CES-D, CGI severity scale, ZAI	Modest therapeutic effect of olanzapine versus placebo on weight gain in outpatients with AN, but no significant benefit for psychological symptoms
Calabrese 2022 Italy	Pilot study	Therapeutic ketogenic diet/ Ketamine infusions	N = 5 SE-AN NR	38.4 > 10	BMI, CIA, EDEQ, Qualitative interviews	TKD followed by ketamine infusion treatment was both safe, effective and helpful in reducing AN-related psychopathology and symptoms. BMI did not significantly change
Mauri 2013 Italy	Case reports	Haloperidol	N = 9 SE-AN-R BMI: 12.2	25.8 7.6	Side effects, BMI, Severity of delusional symptoms	Low-dose haloperidol treatment was well-tolerated and useful in improving BMI, delusional body image disturbance and the drive for thinness in severe and treatment-resistant AN. No side effects reported
Cassano 2003 Italy	Clinical Trial	Haloperidol	N = 13 SE-AN-R BMI: 15.7	22.8 6.3	BMI, EDI, EAT, CGI-S and CGI-I	The use of haloperidol as an addiction therapy in SE-AN-R patients was associated with improvement of ED symptomatology and increased BMI after 6 months of treatment
Israely 2017 Israel	Double-Blind, Randomized Cross-Over Trial	Tyrosine	N = 19 Severe AN BMI: 15.5	22.8 6.3	CTBHPB, EDI-2, BDI, STAI, MPS, LOI	Tyrosine supplementation may improve cognitive function and psychological traits associated with AN (depression)
Van den Eynde 2013 UK	Pilot study	rTMS	N = 10 AN 15,7 NR	25 10	EDE-Q, DASS-21	Diminished feelings of “being fat” or “full” and anxiety
Dalton 2021 UK	Pilot study	rTMS	N = 34 AN ** > **14 NR	NR ** > **3	BMI, Clinical evaluation of depression and anxiety	No differences in regional CBF groups Amygdala-rTMS showed greater increase in weight
Park 2018 USA	Longitudinal trial	DBS at NAcc	N = 6 SE-AN Range 13–16	Range 21–65 > 7	BMI Neuro-psychological/ behavioral measurements	Data will be included in future publications
Martinez 2020 Spain	RCT	DBS at NAcc or SCC	N = 8 SE-AN Range 9,64–16,22	Range 18–60 > 10	BMI Changing in AN behaviors and clinical variants	BMI-RV defined as mean BMI over the last 12 months. After treatment 5 patients had significant BMI and QOL improvement
Dalton 2018 UK	RCT	rTMS	N = 34 SE-AN 16 Caucasian	29,74 14,07	ED symptoms Mood/Psychopathology QOL	rTMS was safe and well tolerated. Small differences in BMI and ED-symptoms on QOL between the groups and moderate results for mood improvement
Bartholdy 2015 UK	RCT	rTMS	N = 44 AN Range 14–18,5	> 18 > 3	EDE-Q + BMI Mood/Psychopathology QOL	Data will be included in future publications
Dalton 2020 UK	RCT	rTMS	N = 30 AN BMI > 14	NR NR	EDE-Q + BMI Mood/Psychopathology QOL	rTMS exerts long lasting effects on mood, but BMI and ED symptom improvement need more time
Dalton 2022 UK	Qualitative study	rTMS	N = 29 SE-AN 16	30.1 15.1	Qualitative interviews	rTMS produced changes in life such as becoming more positive and open‐minded, less anxious, more motivated, more flexible around food and more willing to try new things
Lipsman 2017 USA	Open-label Trial	DBS of subcallosal cingulate	N = 16 SE-AN BMI: 12.3	34 18	BMI, HAMD, BDI, BAI, YBOCS, YBC-EDS, DER, QOL, BIS/BAS, PET imaging	Subcallosal cingulate DBS was safe and well tolerated. It was related with significant and lasting improvements in mood, anxiety, affective regulation, BMI, and changes in neural activity
McClelland 2016 UK	Case series	rTMS	N = 5 SE-AN BMI: 16.1	35.6 20.4	BMI, EDE-Q, DASS-21	rTMS was well tolerated and associated with improvements in ED and affective symptoms that persisted (at least partially) for 12 months
Fernandes Arroteia 2020 Luxembourg	Case report	DBS of NAcc	N = 1 Bulimic SE-AN BMI:12.8	42 > 10	BMI, Subjective QOL	After 1 year of DBS there was a report of impressive weight gain (15 kg) and subjective increase in QOL. No improvement in comorbid depression
Guerrero Alzola 2020 Spain	Case report	Stereotactic cingulotomy	N = 1 AN-R BMI:12.8	46 31	BMI, BSQ, EDI-2, SF-36, TONI-2, BDI-II, HADS, YBOCS	After a 10 year follow-up post stereotactic surgery, the patient is clinically stable with an increased BMI and improved neuropsychological indicators

UK, United Kingdom; USA, United States of America; RCT, Randomized Controlled Trial; CBT, Cognitive- Behavioural Therapy;, SSCM, Specialist Support Clinical Management; IM, Intra Muscular;, rTMS, repetitive Transcranial Magnetic Stimulation; DBS, Deep Brain Stimulation; NAcc, Nucleus Accumbens; BMI, Body Mass Index; AN, Anorexia Nervosa; AN-BP, Anorexia Nervosa Binge eating/Purging; AN-R, AN Restricting subtype; NR, Not Relevant;, SE-AN, Severe Enduring Anorexia Nervosa; BN, Bulimia Nervosa; DD, Disease Duration; EDE, Eating Disorder Examination; GSI, Global Severity Index; BSI, Brief Symptom Inventory; ED, Eating Disorder; EDQOL, Eating Disorders Quality of Life Instrument; MCS, Mental Component Score; BDI, Beck Depression Inventory; SF, Study short Form; ANSOQ, Anorexia Nervosa Stages of Change Questionnaire; EDE-Q, Eating Disorder Examination Questionnaire; EDI-2, Eating Disorder Inventory-2; YBOCS, Yale-Brown Obsessive–Compulsive-Scale; CES-D, Center for Epidemiologic Studies Depression; CGI, Clinical Global Impressions; CGI-S/I, CGI Severity/Improvements; CIA, Clinical Impairment Assessment; ZAI, Zung Anxiety Inventory; EAT, Eating Attitude Test; CTBHPB, Computerized Test Battery for Assessment of Human Performance and Behavior; STAI, State-trait Anxiety inventory; LOI, Leyton Obsessional Inventory; QOL, Quality Of Life; DASS-21, Depression and Anxiety Stress Scales; HAMD, Hamilton Depression Rating Scale; BAI, Beck Anxiety Inventory; YBC-EDS, Yale-Brown-Cornell Eating Disorder Scale; BIS/BAS, Behavioral Inhibition System and Behavioral Activation System; SF-36, 36-Item Short Form Health Survey; TONI-2: test of Nonverbal Intelligence; BDI-II, Beck Depression Inventory; HADS, Hospital Anxiety and Depression Scale.

Eleven studies were randomized controlled trials (RCTs) [26, 28, 32–40], three were pilot studies [41–43], three were longitudinal trials [9, 44, 45], two were quality-assessment studies [46, 47], two were clinical trial [48, 49] and four were case series [50–53].

All participants were female in 14 out of 25 studies, while 11 studies recruited both males and females. The sample size ranged from 1 to 152 participants. The average age ranged from 17 to 65 years. The average ED duration was reported in all the studies we examined, and was approximately 10,94 years (range 3–31 years). Outcome measures varied considerably among studies, ranging from changes in BMI or ED symptoms (both general symptomatology and compensatory and compulsive behavior) to changes in mood and quality of life.

The three macro-themes we identified are the following: 1.“Psychotherapy and Specialist Supportive Clinical Management”, 2. “Pharmacological Treatments” and 3. “Brain Stimulation Therapies”. They are described in the following sections.

### Theme 1: Psychotherapy and specialist supportive clinical management

The effect of psychotherapy on patients affected by SE-AN was evaluated by six of the studies included in this review. An RCT conducted by Dalle Grave described the application of Cognitive Behavioral Therapy (CBT) in inpatients with SE-AN [37]. The treatment consisted in individual and group CBT-E sessions. This study showed that inpatient CBT-E is well accepted by patients with severe AN and the response is promising. 90% of the patients completed the program, and most improved substantially. Deterioration after discharge occurred but was not severe or long-lasting.

In a longitudinal outcome study, Calugi et al. aimed to assess short and long-term outcomes in SE-AN patients, compared with non-SE-AN (NSE-AN) patients, both treated using a “recovery model” approach based on inpatient Enhanced Cognitive Behavioral Therapy for ED (CBT-E) [9]. More than 80% of eligible patients agreed to undergo the psychological treatment, and among them 85% completed it. A significant increase in BMI, and substantial improvements in ED-specific and general psychopathology in both NSE-AN and SE-AN groups were achieved by inpatient CBT-E. No significant differences were found in short or long-term outcomes of inpatient CBT-E between NSE-AN and SE-AN patients. These results show that a recovery model approach, such as inpatient CBT-E, is well accepted by AN patients, and could also be a valuable and promising treatment for those with SE-AN, as long as they are fully committed.

The quality assessment study by Frostad et al. included 21 patients between the ages of 17 and 51 with severe or extreme AN (BMI < 16 kg/m2) [46]. All enrolled patients received CBT-E to treat the ED. Their BMI was measured at baseline, at the end of CBT-E and one year after the end of treatment. Almost half of the patients that started treatment completed it; the remaining 52.4% prematurely dropped out. In the group of patients that completed the therapy there was significant weight gain at the end of the treatment (EOT). These results confirm those of previous studies, which indicate that CBT-E may be suitable for patients with severe and extreme AN without acute medical complications.

In the RCT by Touyz et al., two psychological treatments specifically adapted for chronic patients were compared: cognitive behavioral therapy (CBT-E) and Specialist Supportive Clinical Management (SSCM-SE), in the form of education, care and support aimed at assisting patients through the use of praise, reassurance and advice [32, 54]*.* Weight gain was actively promoted, but the primary goal was to improve quality of life. Both treatment methods were successful in promoting such a change, but in a 12-month follow-up period, patients who received CBT-SE obtained globally lower scores in the Eating Disorder Examination (EDE) and greater social adjustment compared to patients receiving SSCM-SE [55]. These results suggest that refocusing (?) treatment goals while maintaining a focus on quality of life and harm reduction could ultimately be a better approach for people with SE-AN than approaches that define remission as exclusively based on weight regain and elimination of the ED.

Le Grange et al. aimed to identify predictors and moderators of outcome at the end of treatment (EOT) and at 6 and 12-month follow-up for adults with SE-AN in an RCT study [40]. In this moderator analysis of treatment outcome, CBT-AN was found to be more beneficial than SSCM when patients had worse depression levels (as measured on the BDI), or older age, or more severe ED-related symptoms, or suffered from AN-BP subtype (binge eating/purging). Although it is useful to have identified a patient subgroup for which CBT-AN is more beneficial, it was disappointing not to have found one for which SSCM is the recommended therapy.

In the Randomized Controlled Trial by Elbaky et al., a total of 63 participants were randomly assigned to CBT-AN, which makes use of specific cognitive and behavioral strategies, or SSCM, described as a more collaborative and supportive therapeutic style [36]. Participants had severe and enduring illnesses and very poor health-related quality of life. There were no significant differences between the two treatment groups at treatment completion (?).

### Theme 2: Pharmacological treatments

Seven studies included in this review evaluated pharmacological treatments. The drugs studied were dronabinol (one study), ketamine (two studies), olanzapine (one study), haloperidol (two studies) and tyrosine (one study).

Andries's randomized controlled trial specifically selected only people with SE-AN (AN lasting for more than five years as an inclusion criterion), aged between 18 and 25 years, and investigated the orexigenic and anabolic effects of dronabinol[56]. Dronabinol is a synthetic cannabinoid that, as well as improving appetite through the endocannabinoid system, also appears to have anabolic effects by interacting with molecular hubs involved in the peripheral fat metabolism [28, 57]. Participants received 2,5 mg of dronabinol twice a day for four weeks and a matching placebo for four weeks, separated by a four-week washout period. Despite small weight improvements in the dronabinol group, changes in ED symptoms were minimal and did not differ between the two groups. No serious adverse events were reported in either group, and the side effects reported were similar in the dronabinol and placebo groups.

Ketamine was evaluated in a longitudinal study that included patients with long-term AN or BN in comorbidity with treatment-resistant depression (TRD) [44]*.* IM ketamine (dose 0.5–0.80 mg/kg), administered with repeated dosing at four-to-six-week intervals, resulted in clinically significant changes in depression and, to a lesser extent, in anxiety and ED symptoms. This pilot study concluded that IM ketamine is effective for TRD in patients with severe and long-lasting ED. However, ketamine effects on ED symptoms were modest. The role of ketamine in the management of SE-AN has also been assessed in a pilot study by Calabrese et al. [43]. Five female adults whose weight had recovered from AN, but who had persistent ED-psychopathology, were given a Therapeutic Ketogenic Diet (TKD) directed towards weight maintenance and nutritional ketosis, designed by an experienced ketogenic dietitian which involved a two-day immersion program and home maintenance of nutritional ketosis for four-to-eight weeks. Once the participants had sustained nutritional ketosis, they were administered six titrated ketamine infusions and were followed up for six months. No significant adverse effects emerged throughout the duration of the study protocol. Two participants maintained TKD for eight weeks before the ketamine infusions due to a good behavioral response. The findings of this study suggest that TKD, which aims to establish and maintain nutritional ketosis but not weight loss, followed by ketamine infusion treatment is both safe, effective, and able to improve symptomatology in subjects with AN who have regained weight but still have serious ongoing AN-related preoccupations, such as fear of weight gain and concerns around body shape and self-acceptance. Further studies are needed to determine whether TKD or ketamine have specific effects on depression that subsequently improve AN-related psychopathology, or whether the effects are independent.

Other pharmacological treatments that may contribute to the management of SE-AN patients have been evaluated over time. In the study by Attia et al. the benefits of olanzapine versus placebo for adult outpatients with anorexia nervosa were assessed [26]. In this randomized, double-blind, placebo-controlled 16-week trial, 152 adult outpatients with AN were enrolled. Olanzapine was well-tolerated. This study revealed a modest therapeutic effect of olanzapine versus placebo on weight gain in outpatients with AN, but no significant benefit on psychological symptoms.

The clinical trial by Cassano et al. aimed to evaluate the effectiveness of haloperidol (a selective D2 receptor blocker) as an adjunctive treatment for treatment-resistant anorexia nervosa, restrictive subtype (AN-R) [49]. In this trial, a selected sample of 13 female outpatients with treatment-resistant AN-R were treated for six months with low-dose haloperidol (1–2 mg/day) in addition to standard treatment. The treatment was associated with a significant overall improvement of ED symptomatology and an increase in BMI after six months, suggesting a potential therapeutic role in this specific population. The case series by Mauri et al. [51] assessed the therapeutic potential of low-dose (between 0.5 and 3.3 mg/day) haloperidol in nine female adult inpatients suffering from severe treatment-resistant AN, characterized by a mean BMI < 13 kg/m^2^ and a delusional body image disturbance. All participants became less concerned about weight gain, and they subjectively perceived decreased intensity both of the urge for thinness and of the delusional body image disturbance.

Israely et al.’s double-blind randomized cross-over trial evaluated the effect of oral tyrosine administration on the cognitive function and emotional-psychological state of severe inpatients suffering from AN [39]. Tyrosine is an essential amino-acid precursor of catecholamines (CA) that has been found to be reduced in AN, and it may contribute to behavioral-psychologic disorders and thus to a vicious cycle of dieting and weight loss [58]^59^, ^60^. The authors hypothesized that the lack of essential dietary-derived neurotransmitter precursors could be a part of the complex psychological and emotional disturbances involved in AN. They imagined that tyrosine supplementation could improve psychological traits associated with AN (such as mood) and cognitive function without affecting body weight, therefore facilitating nutritional rehabilitation. Apparently, tyrosine supplementation affects memory-related performance, as similar studies that administered tyrosine to healthy subjects have found [61]. However, in this study no significant results were found on weight restoration and BMI. A decrease in depression scores following tyrosine supplementation is consistent with findings from previous studies that showed a beneficial effect of tyrosine administration on depression in both healthy persons and patients [62, 63]. Tyrosine supplementation may improve psychological traits associated with SE-AN, such as depressive mood, and cognitive function (it reduced test duration and time of reaction in memory tasks), but future studies need to be conducted on a large scale to assess whether it can be a useful treatment in the management of patients with AN.

### Theme 3: Brain-directed therapies

Seven studies assessed Repetitive Transcranial Magnetic Stimulation (rTMS). The first study conducted to investigate the therapeutic potential of rTMS in SE‐AN found that rTMS treatment was feasible, safe, and well‐tolerated [33, 42]. Participants assigned to real rTMS showed marked improvements in mood, medium improvements in quality of life and small changes in BMI, compared to the sham rTMS group [33]*.* In‐depth semi‐structured qualitative interviews were conducted to evaluate the treatment experience of rTMS in people with SE‐AN, with the aim of systematically investigating participants' opinions, hopes, worries and expectations about rTMS treatment. rTMS was found to be an acceptable but time‐consuming treatment. Many pointed out that their lives changed after treatment, because it had made them more positive and open‐minded, less anxious, more motivated, more flexible around food and eating and more willing to try new things both in areas related to their AN and in other parts of their lives [34, 47]. These results also offer a potential support to previous neuroimaging findings [41]. Specifically, they suggest that the reported decrease in amygdala activity (during the period of rTMS treatment) could be associated with longer‐term weight gain as a result of an improved ability to tolerate unpleasant emotional and physical sensations (e.g., anxiety/fear of food). The therapeutic potential of repetitive transcranial magnetic stimulation (rTMS) in people with enduring AN was also examined in a case series by McClelland et al. [50]. From pre-treatment to post-treatment, BMI did not substantially change, while ED symptoms and general psychopathology improved significantly. Participants’ qualitative feedback regarding the intervention was substantially positive and encouraging. Most reported improved motivation towards recovery, coping ability and affect regulation after rTMS. However, most participants had lost some weight (slight decrease in BMI), and therapeutic effects on ED psychopathology had moderately reduced at 12-month follow-up.

Four studies evaluated Deep Brain Stimulation (DBS) of the Subcallosal Cingulate and Nucleus Accumbens. Lipsman reviewed safety, clinical outcomes and neuroimaging (PET scan) outcomes of subcallosal cingulate-DBS in SE-AN patients analyzed over 12 months of active stimulation [48]. The DBS safety profile in SE-AN was not substantially different from that observed in other studies that used DBS as a treatment for psychiatric disorders, such as OCD or depression. In addition, there were significant and lasting improvements in mood, anxiety, affective regulation, BMI, and changes in neural activity at the end of the follow-up period: this means that DBS can impact the natural course of SE-AN and can directly influence AN-related brain circuits. The potential therapeutic role of DBS in SE-AN was also assessed in a case report by Arroteia et al., where they described impressive weight gain in a 42-year-old woman suffering from chronic and treatment-resistant AN (bulimic subtype) after 12 months of bilateral Nucleus Accumbens (NAcc) DBS [52]. DBS of the NAcc is a treatment option that should be considered in SE-AN when conventional treatment approaches recommended by evidence-based guidelines have not been able to ensure long-lasting improvements in ED-related symptoms and psychopathology. The RTC conducted by Martinez reported significant improvements in BMI and weight gain, but not in ED symptoms and psychopathology [35]. In order to study the application of DBS research in individuals with SE-AN, Park et al*.* have presented an innovative clinical trial protocol, which is still ongoing [64].

Only one study assessed a brain-centered intervention, stereotactic surgery, that cannot be formally stated as a brain stimulation therapy because of its ablative purpose (which differs from DBS stimulating surgery). In Alzola’s study, marked improvements in neuropsychological indicators of anxiety, depression and subjects’ perceived quality of life were observed [53]. The patients refused any type of psychotherapy. For this reason, the improvements recorded may be attributed almost exclusively to the surgical intervention. Stereotactic surgery may be an option for patients with chronic anorexia nervosa where conventional treatments have proved insufficient.

## Discussion

AN presents clinicians with significant challenges due to its chronicity, high relapse risk, and considerable morbidity and mortality rates [5–8]. The ego-syntonic nature of the disorder, characterized by denial and lack of treatment motivation, distinguishes AN from other mental disorders [3, 4]. Despite limited scientific evidence on SE-AN treatments, the future holds encouraging prospects for developing more effective therapeutic approaches [19, 20]. The present review examined various treatment options for individuals with SE-AN; in particular, psychotherapeutic, pharmacological, and neurostimulation approaches were identified.

Various reports on treatment effectiveness in individuals with chronic AN evaluate CBT and its greater effectiveness compared to SSCM [9, 32, 36, 40, 65]. Similarly, several studies have reported that inpatient CBT-E is well-accepted and effective [9, 32, 36, 37]. Only the more recent study by Frostad et al*.*, regarding the therapeutic potential of outpatient CBT-E, reported an elevated dropout rate, suggesting that CBT is more tolerated when carried out in an inpatient setting [46]. Nonetheless, the group of patients that completed the treatment achieved significant weight gain both at EOT and after a one-year follow-up (80% had a BMI ≥ 18.5), proving that outpatient CBT-E is a valid alternative to hospitalization [46]. Le Grange et al. identified predictors and moderators of outcome at the end of treatment: worse depression level, older age, more severe ED-related symptoms, and AN-BP subtype (binge eating/purging) [40].

When considering pharmacological treatments, research on dronabinol, antipsychotics, and ketamine showed some mixed results regarding the often complementary areas of weight gain and improvement in ED-related symptoms [26, 43, 44, 49, 51, 56]. Some medications showed positive effects on weight gain [26, 43, 49, 51, 56], and others on ED psychopathology [43, 44, 49, 51]. Dronabinol, a synthetic orexigenic cannabinoid, showed some modest effects on weight gain and metabolic changes [56]. Conversely, it was found to be ineffective in improving ED psychopathology. Such a profile could hold clinical importance for patients with SE-AN even in the absence of notable changes in psychopathological symptoms [56].

Regarding antipsychotics, some studies showed that olanzapine and haloperidol can have the potential to induce weight gain [26, 49, 51]. These results, however, show some contradictions, such as the fact that olanzapine offered modest benefits in weight gain, considerably less than the substantial weight gain observed in treating other disorders using the same pharmacological therapy [26]. Moreover, evidence for olanzapine's significant impact on AN-psychopathological features, such as excessive concerns about weight gain and obsessiveness, is conflicting [26, 66]. Regarding haloperidol, some small studies showed how low-dose inpatient treatment exhibited improvements in BMI and ED psychopathology, particularly in delusional symptoms and drive for thinness [49, 51].

With respect to ketamine, some studies indicated promising treatment approaches for SE-AN and its comorbid conditions. In fact, research showed that intramuscular ketamine had a beneficial effect on depressive symptoms, revealing high efficacy in reducing depressive symptoms and the risk of suicide [44]. Minor improvements were observed in controlling anxiety and ED-related symptoms [44]. It was also demonstrated that combining ketamine infusions with a therapeutic ketogenic diet can be safe and effective in reducing ED-related symptoms and psychopathology, despite a lack of significant weight restoration [43].

Combining TKD with ketamine may help normalize impaired dopaminergic function in AN, potentially improving brain circuit function and behaviors. Although the underlying mechanisms remain unclear, the results suggest that this approach is effective in AN patients who have restored weight but continue struggling with body image concerns and self-acceptance issues [67–69]. Similarly, with regard to nutritional supplements, tyrosine has shown potential therapeutic efficacy in improving psychological traits associated with AN, such as depressed mood and cognitive function [58, 59, 70, 71].

Finally, on the subject of brain stimulation therapies, such as repetitive rTMS and DBS, we found promising results in improving ED-related psychological traits (such as mood and anxiety), affective regulation, and quality of life [34, 42, 47, 48, 52]. However, we have observed divergent results regarding outcome measures such as BMI and weight gain. Some authors reported significant improvements in this regard [35, 48, 52, 53], while others described small ones, if any [33, 34, 53]. Similarly, discordant findings were reported about ED symptoms, with some moderately positive results [42, 48, 50, 53] and some not significant findings [33–35].

In particular, the use of rTMS on DLPFC seems to hold potential therapeutic effects. Its action in this cortical area can lead to emotional regulation, cognitive flexibility, and response inhibition [72–75]. Even in this field, research shows conflicting results regarding ED psychopathology, weight, and food intake: some studies reported no clinical improvement, while others observed reductions in food cravings and binge eating episodes [50]. Moreover, some authors suggested that rTMS is most effective as an addition to psychological therapy and/ or cognitive training [76–78].

On the other hand, stereotactic neurosurgery acts through neuromodulation of targeted limbic structures such as the subcallosal cingulate [48, 79–81]. The two main types of stereotactic neurosurgery are simulating surgery, involving DBS, and ablative surgery, by means of thermocoagulation and radiosurgery. Stereotactic neurosurgery for psychiatric disorders aims to treat specific symptoms like anxiety, aggression, obsession and compulsion by interrupting neural circuits in the limbic system through surgical blocks using functional neuroimaging, particularly tractography. Chronic DBS stimulation in these regions lead to reduced activity near the target and hyperactivity of parietal structures involved in the limbic and parietal regions, thus leading to impairments in affective functioning [80]. These results were found in both patients with the active disease and those who recovered and restored weight [82, 83]. The cingulate in fact plays a part in attributing reward value to environmental stimuli and affective processing, both of which are impacted in SE-AN [84]. Recent research has also explored neurosurgical procedures like anterior capsulotomy for obsessive symptomatology and anterior cingulotomy for anxious symptomatology [85, 86].

By acknowledging the patient as the central figure in the therapy, interventions should involve intrapersonal work, collaboration with significant others, and identity development beyond the confines of Anorexia Nervosa. Factors identified by Starzomska et al., including enhanced insight, externalization and devaluation of the disorder, and improved interpersonal understanding, play crucial roles in fostering self-determined recovery efforts [87]. Furthermore, therapeutic strategies should extend beyond towards the patient’s interpersonal relationships, involving caregivers and family members, in order to embrace the recent Maudsley AN treatment approach in adults [88]. This approach implies intrapersonal exploration, collaboration with significant others, examination of identity beyond AN, questioning the value of AN, and externalizing its impact on the individual's life.

## Limitations

Research evidence on treatment options for SE-AN remains limited, and the methodological quality of studies is generally low. This review of treatment options for SE-AN highlights the limitations of current studies, including heterogeneity in sample size, follow-up periods, and treatment methods. The definition of SE-AN remains unclear and changes among the different studies. On the basis of the search string set, deliberately kept broad with the desire to offer a wide range of therapeutic modalities, some relevant studies on the topic may have been excluded. It remains of fundamental importance for the future to offer a review of the literature specifically focused on the individual therapies offered for SE-AN, with due precision and in-depth analysis.

## Conclusions

In conclusion, several of the studies reviewed reported effectiveness of CBT treatments for individuals with chronic AN, showing greater effectiveness compared to SSCM, to the point of demonstrating that outpatient CBT is a valid alternative to hospitalization. Regarding the pharmacological treatments analyzed, a very great heterogeneity of results emerges. In particular, some drugs have shown positive effects on weight gain, such as dronabinol and antipsychotics, others on the psychopathology of ED, in particular ketamine. With respect to brain stimulation therapies, such as repetitive rTMS and DBS, we have found promising results in improving psychological traits, affective regulation, and quality of life. An integrated and personalized therapeutic approach adapted to individual needs is vital to manage the complexity of SE-AN, which includes various psychopathological elements, physical alterations, and often multiple psychiatric comorbidities. Embracing such an integrated and patient-centered approach can give rise to new and refined treatment strategies that effectively address the multiple challenges posed by SE-AN.

## Data Availability

Not applicable.
